# Risk Factors of Fatal Outcome in Patients With COVID-19 Pneumonia

**DOI:** 10.1017/dmp.2020.346

**Published:** 2020-09-10

**Authors:** Michaela Cellina, Daniele Gibelli, Carlo Valenti Pittino, Tahereh Toluian, Pietro Marino, Giancarlo Oliva

**Affiliations:** Department of Radiology, ASST Fatebenefratelli Sacco, Milano, Italy; Dipartimento di Scienze Biomediche per la Salute, Università degli Studi di Milano, Milano, Italy; Scuola di Specializzazione in Radiodiagnostica, Università degli Studi di Milano, Milano, Italy; Department of Emergency Medicine, ASST Fatebenefratelli Sacco, Milano, Italy

**Keywords:** coronavirus disease, COVID-19, patient outcome assessment, pneumonia, radiography, viral

## Abstract

**Objectives:**

The aim of this study was to correlate the clinical, laboratory, and radiographic characteristics of patients with a confirmed diagnosis of coronavirus disease 2019 (COVID-19) disease, with fatal outcome.

**Methods:**

We reviewed chest X-ray (CXR) features, clinical, and laboratory data of patients with reverse transcriptase polymerase-chain-reaction confirmed diagnosis of COVID-19 infection. The relationship with mortality was investigated by fitting a logistic regression model.

**Results:**

A total of 246 patients were included (170 males; mean age, 63 y). Most of the patients had 1 or more comorbidity (62%); fever (95%), and cough (60%) were the most common symptoms; CXR detected abnormalities in 88.6%, mainly showing ground-glass opacities (GGO) (90%) with bilateral (64%) and peripheral (46%) distribution.

Multivariate analysis showed that age (*P* < 0.001; mortality of 59% in patients >66 y old; 5% at a younger age) and consolidation at CXR (*P* = 0.001; mortality of 11% with positive CXR; 2% in those without) represented the 2 most significant independent risk factors of mortality. Chronic pathologies, such as diabetes and chronic obstructive pulmonary disease, and peripheral GGO at CXR also showed a significant correlation with mortality.

**Conclusions:**

We identified predictive factors for the fatal outcome of COVID-19 patients. The prognostic value of these findings can be useful for optimal patient management and resource allocation.

Several cases of pneumonia of unknown etiology have been reported in Wuhan City, Hubei Province, China, in December 2019.^1^ The virus causing the epidemic was identified on January 7 as a new coronavirus (2019-nCoV), and the resulting pneumonia was named by the WHO as coronavirus disease 2019 (COVID-19).^2^ As of August 31, 2020, a total of 25,118,689 confirmed cases and of 844,312 deaths have been reported worldwide.^3^

Clinical manifestations of COVID-19 infection are highly variable: serious cases develop severe pneumonia, acute respiratory distress syndrome (ARDS), and multiple organ failures leading to death; nonsevere cases present ordinary symptoms of respiratory system infection; and asymptomatic cases have also been reported.^2,4-7^

One of the issues the involved countries are facing is represented by the very high volume of patients presenting to health centers or hospitals during the outbreak, that overwhelms the health-care resources available, especially the need for critical care support.

Early and effective predictors of clinical outcomes are urgently needed for risk stratification of COVID-19 patients, to help effective patient management and resource allocation.^8,9^

Therefore, our aim was to retrospectively analyze clinical, laboratory, and radiographic characteristics of a consecutive series of patients who presented to our emergency department (ED) with symptoms suspected for COVID-19 infection, with confirmed diagnosis by real-time reverse transcriptase polymerase chain reaction (RT-PCR), to assess the correlation with fatal outcome and to identify variables with prognostic value.

## METHODS

This single-center retrospective study was approved by our institutional review board and performed in a hospital with approximately 60,000 annual ED accesses, located in the center of Milan, in Northern Italy, an area heavily hit by the COVID-19 outbreak. Consent was obtained from the patients involved: after confirmation of COVID-19 positivity, the patients were asked to provide consent regarding the use of anonymized demographic and clinical information and submission to the experimental treatments.

All clinical, laboratory, and outcome data were extracted from electronic medical records using a standardized data collection form.

### Patients

We retrospectively analyzed data from our hospital ED electronic database of consecutive patients who presented to our ED with symptoms suspected of COVID-19 infections from 15 February to 30 March 2020.

Inclusion criteria were as follows: (1) patients aged more than 18 y, as our hospital was converted to a COVID-19 hub for adults, whereas pediatric patients were treated in other designated institutions; (2) COVID-19 diagnosis confirmation through RT-PCR test performed using nasal and rhinopharyngeal swab specimens; (3) availability of chest X-ray (CXR) and laboratory blood tests executed at patient arrival, and of clinical (symptoms and comorbidities) data assessed at patient admission; (4) patient discharged or deceased.

The clinical outcomes of recovery or death were monitored up to 30 April 3 2020, the final date of follow-up.

### Clinical Data

All data were collected in a standardized Excel electronic database; the variables to insert were decided in consensus by an emergency physician and a radiologist in a preliminary meeting, mainly based only upon our clinical practical experience.

Clinical data and radiological images collection were performed by 2 radiology residents in consensus, blinded to the aim of the study, trained in previous research studies. Data recording was made under the supervision of a radiologist, who checked the correctness of the information.

Patients with missing data were excluded from the analysis. Weekly meetings were held between all study participants to check the progress of data collection.

The following comorbidities were investigated from patients’ history: diabetes, arterial hypertension, chronic obstructive pulmonary disease, asthma, cardiovascular disease, cardiac failure, atrial fibrillation, stroke, dementia, obesity, chronic renal insufficiency, malignancy, HIV infection (according to the patients’ characteristics collected by the Epidemiology for Public Health-Istituto Superiore di Sanità.^10^ The assumption of angiotensin-converting enzyme inhibitors (ACEi) was recorded, as ACE2 has been shown to be a co-receptor for viral entry for severe acute respiratory syndrome coronavirus 2 (SARS-CoV-2), with increasing evidence of its role in the pathogenesis of COVID-19, and a concern that the use of ACEI increases expression of ACE2 and patient susceptibility to viral host cell entry and propagation.^11,12^

We collected the following patient symptoms: cough, dyspnea, hemoptysis, chest pain, cutaneous rash, gastrointestinal symptoms, conjunctivitis, fever, and days of fever. Temperature and oxygen saturation at patient arrival were collected, as well as patients’ treatments and days of hospitalization.

### Blood Tests

We collected the following blood tests, according to the protocol adopted for suspected COVID-19 patients in our clinical practice, and to the agreement between the emergency physician and the radiologist who decided the variables to collect: leukocytes, neutrophils, lymphocytes, platelets, erythrocytes, D-dimer, aspartate aminotransferase, alanine aminotransferase, lactate dehydrogenase, C-reactive protein, fibrinogen, troponin T, arterial blood gases (pH, PaO2, PaCO2, SaO2). Creatinine was not included, as we already considered chronic renal insufficiency as a variable.

### X-Ray Analysis and Quantification

All CXRs were acquired in the posteroanterior (PA) + laterolateral projections on a fixed radiographic machine, or in the anteroposterior (AP) projection on a portable radiographic unit, according to our standard acquisition protocols.

**FIGURE 1:**
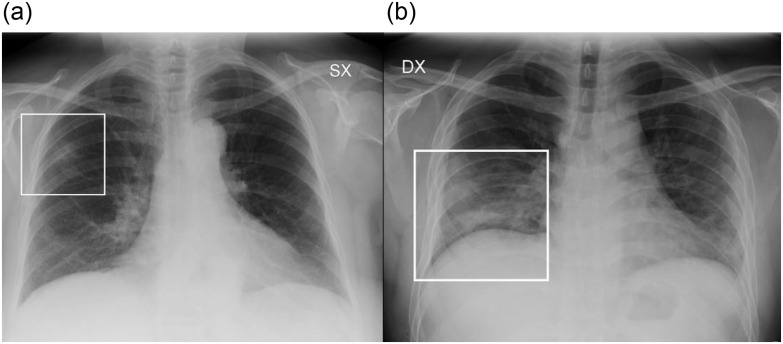
Examples of CXR Abnormalities. a: CXR showing a focal GGO involving the middle-upper fields of the right lung (frame). b: CXR showing area of consolidation in the lower right fields (frame). GGO are recognizable in the lower fields of the left lung.

**FIGURE 2:**
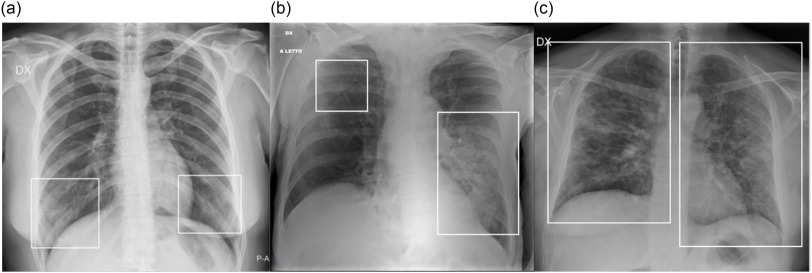
Examples of CXR Severity Score Assignment. a: CXR showing focal bilateral GGO in the lower fields (frames). On both left and right lung, the involvement was < 25%; therefore, the CXR severity score assigned was 1 for each lung, with a global score of 2. b: CXR showing bilateral parenchymal opacities (frames): a huge area of consolidation in the middle-lower left fields with contextual air bronchogram, while a focal area of GGO is recognizable in the upper fields of the left lung; the extension on the left side was > 50% (score 3), whereas the involvement on the right side was < 25% (score 1); therefore, the overall score was 3 + 1 = 4. c: CXR showing bilateral involvement, with mixed areas of GGO and consolidation (frames) involving all the lung fields. On both left and right lung, the involvement was > 75% (score 4); therefore, the global score was 4 + 4 = 8.

Two radiologists (M.C., a radiologist with 9 y of experience; M.O., a radiologist with 7 y of experience) in consensus assessed for each CXR: (1) the presence of lung abnormalities, described as consolidation, ground-glass opacities (GGO), or nodules, according to the Fleischner Society glossary of terms^13^ (Figure 1); (2) their distribution, classified into (i) “peripheral,” “central,” or “both”; and into (ii) “unilateral” or “bilateral.” The presence of pleural effusion was recorded.

A radiographic severity score, according to Wong et al.,^14^ was independently assigned by 2 radiologists (M.P., radiologist with 7 y of experience; G.O., radiologist with 25 y of experience), depending on the extent of involvement by consolidation or GGO (0 = no involvement; 1 = <25%; 2 = 25-50%; 3 = 50-75%; 4 = >75% involvement), for each lung, with a maximum score of 8 for CXR. Some examples are provided in Figure 2. Interobserver agreement was evaluated.

### Statistical Analysis

Values were checked with a 1-sample Kolmogorov-Smirnov test for normality. Interobserver agreement was assessed through Cohen’s k correlation coefficient. Kruskal-Wallis H test and Mann-Whitney U-test were used to evaluate differences between independent groups. Chi-squared test or Fisher’s exact test were used to determine significant relationships between categorical variables. Correlations between values were evaluated through Spearman’s Correlation Coefficient: among redundant variables (Spearman coefficient > .80). The relationship between fatal outcome with CXR severity score, radiological features, and clinical and laboratory parameters was investigated fitting a logistic regression model. *P* < 0.05 was regarded as statistically significant. To avoid redundancy, we selected between all variables with *P* < 0.05 the independent variables (Spearman’s K < 0.8) and the most informative among redundant variables, based on receiver operating characteristic analysis. A model was built with the variables thus selected by a backward stepwise model. A chi-squared automatic interaction detection (CHAID) decision tree analysis with CXR score as the user-specified first level was used to detect the fatal outcome. Statistical analysis was performed using SPSS (IBM SPSS Statistics, Version 26.0, IBM Corp).

## RESULTS

### Patients

According to our exclusion criteria, 246 patients were enrolled. Most patients were men (170/246; 69%), with a mean age of 63 y; 154/246 patients (62%) have at least 1 chronic disease (Table 1). Upon admission, most patients had fever (234/246; 95%) and cough (148/246; 60%); C-reactive protein levels were increased in the 216/246 (88%) of patients (Table 2).

**TABLE 1:** Role of the PCSS in Identifying Issues and Solutions

Issue	Group Affected	PCSS Action	Result
Concern about discharging patients with high oxygen requirements with exertion	Patients with oxygen levels >2 L/min with exertion	Group coordination to develop a protocol involving multiple groups	New Home Oxygen Pathway with specific attention to this group of patients
Patients unable to discharge to care facilities due to COVID-19	Patients no longer meeting inpatient criteria but unable to return home	Group coordination to determine what care facilities would deem acceptable acceptance criteria	Negative COVID-19 nasopharyngeal swabs X2 was considered acceptable risk and patients then were transferred successfully to care facilities
Lack of standardization in discharge care for patients with COVID-19	All COVID-19 positive inpatients	Group coordination between extensive stakeholders including outpatient providers, telehealth monitoring, and respiratory therapy	Standardized discharge pathway easily orderable through the EMR with the additional benefit of standardized “dot phrases” allowing standardized verbiage in the chart and for home-going
Respiratory therapy overwhelmed by need for home oxygen to be completed coupled with increase in tracheostomy care and complex respiratory issues	Respiratory therapy	Group creation of a new group of back-up respiratory therapists	Allows main respiratory therapists to provide direct patient care while back-up therapists completed the challenging home oxygen process

**TABLE 2:** Patients Signs, Symptoms, and Laboratory Tests and Their Correlation With Fatal Outcome

Sign and Symptoms	Patients Number/total (%)	Statistical correlation with fatal outcome (*P*)
Cough	148/246 (60.1%)	0.140 (Pearson X2 test)
Dyspnea	120/246 (48.8%)	0.001 (Pearson X2 test)
Hemoptysis	2/246 (0.8%)	0.656 (Pearson X2 test)
Chest pain	12/246 (4.9%)	0.336 (Pearson X2 test)
Cutaneous rash	2/246 (0.8%)	0.656 (Pearson X2 test)
Gastrointestinal symptoms	52/246 (21.14%)	0.722 (Pearson X2 test)
Conjunctivitis	4/246 (1.63%)	0.527 (Pearson X2 test)
Fever	234/246 (95.1%)	0.336 (Pearson X2 test)
Days of fever	6.7±6 (3.5%)	<0.001 (Pearson X2 test)
Temperature	37.9±0.8° C	0.432 (Mann-Whitney U-test)
Oxygen saturation	91.5±7.7	<0.001 (Mann-Whitney U-test)
**Blood tests**		***P*** **(Mann-Whitney U-test)**
Leukocytes	7.3±3.9 10^9^/L (NR 4.19-9.35 10^9^/L)	0.023
Neutrophils	5.4±3.9 10^9^/L (NR 1.91-6.23 10^9^/L)	0.126
Monomorphous **%**	24.87±13 %	0.004
Polymorphus **%**	75.15±12.9 %	0.004
Lymphocytes	1.4±1.4 x10^9^/L (NR 1.13-3.37x10^9^/L)	0.028
Platelets	227±111x10^9^/L (NR. 169-359 10^9^/L)	0.463
Erythrocytes	4.7±0.8x10^12^/L (NR 4.13-5.15*10^12/L)	0.084
D-dimer	1532±5600 ng/mL (NR 250-500 ng/mL)	<0.001
Aspartate aminotransferase	54.1±38.2 U/L (NR 10-35 U/L)	0.290
Alanine aminotransferase	44.6±41 U/L (NR. 10-33 U/L)	0.455
Lactate dehydrogenase	353.6±148.9 U/L (NR 135-225 U/L)	0.023
C-reactive protein	99.8±94.3 mg/L (NR 0-5 mg/L)	<0.001
Fibrinogen	633.86±88.82 (V.N. 270-470 mg/dL)	0.007
Troponin T	32.03±38.29 (V.N. 0-14 ng/L)	0.001
Arterial blood gas	pH 7.46±0.056 PaO2 63.68±18.85 PaCO2 33.40±8.94 SaO2 91.16±11.29	0.381 0.620 0.951 0.443

Abbreviation: NR, normal range.

Most of the patients received a combination of antibiotics and antiviral therapy (152/246 patients; 62%). Patients’ treatments are listed in Table 3.

**TABLE 3:** Patients Treatment During Hospitalization

	Patients Number/total (%)
Non-invasive ventilation	86/246 (34.9%)
Invasive ventilation	26/246 (10.5%)
Hydroxychloroquine	206/246 (83.7%)
Antiviral treatment (lopinavir/ritonavir)	166/246 (67.5%)
Antibiotic treatment (Azithromycin)	218/246 (88.6%)
Tocilizumab	46/246 (18.7%)
Paracetamol	148/246 (60.1%)
Dexamethasone	88 (35.7%)
Methylprednisolone	16 (6.5%)

### CXR Analysis and Quantification

One hundred fifty-six of 246 (63%) CXR were executed in the posteroanterior projection on a portable X-Ray unit. Two hundred eighteen of 246 (88.6%) of CXR showed abnormalities. Most patients (222/246; 90.2%) showed GGO, with bilateral (158/246, 64.2%) and peripheral (114/246, 46.3%) distribution. The mean radiographic severity score was 3 ± 2. Interobserver agreement was excellent (Cohen’s K coefficient: 0.901). Overall imaging findings are listed in Table 4.

**TABLE 4:** Radiographic Findings and Their Distribution, and Radiographic Severity Score in Our Patient Population and Correlation With the Fatal Outcome

	Patients number/total (%)	Statistical correlation with fatal outcome (*P*)
**Presence of X-ray abnormalities**	218/246 (88.6%)	< 0.001 (Pearson X2 test)
**Radiographic findings**		**Pearson X2 test**
GGO	222/246 (90.2%)	< 0.001
Consolidation	106/246 (43%)	< 0.001
Nodules	0/246 (0%)	
Pleural effusion	26/246 (10.5%)	< 0.001
**Distribution**		**< 0.001 (Pearson X2 test)**
Peripheral	114/246 (46.3%)	
Central	30/246 (12.2%)	
Peripheral+central	80/246 (32.5%)	
**Side**		**< .001 (Pearson X2 test)**
Unilateral	66/246 (26.8%)	
Bilateral	158/246 (64.2%)	
**Severity Score**	3.28±2.02	**< .001 (Pearson X2 test)**

Abbreviation: GGO, ground glass opacity.

### Fatal Outcome Prediction

#### Univariate Analysis

The correlation upon univariate analysis of the analyzed variables with fatal outcome is listed in Tables 1, 2, 4.

We observed that different variables significantly correlated with the outcome: sex (*P* = 0.001), and age (*P* < 0.001), various comorbidities, assumption of ACEi (*P* < 0.001), and days of hospitalization (*P* < 0.001).

Analyzing symptoms upon admission, only dyspnea correlated with mortality (*P* = 0.001); fever did not correlate (*P* = 0.336), but the days of fever did (*P* < 0.001).

Among blood tests, C-reactive protein, D-dimer, and fibrinogen were significantly correlated with the outcome (*P* = 0.001).

At CXR, GGO with peripheral distribution, consolidation, pleural effusion, and the severity score showed a significant correlation with fatal outcome (*P* < 0.001).

#### Multivariate Analysis

We confirmed the significant correlation with fatal outcome of age (*P* < 0.001; odds radio [OR], 1.206; 95% confidence interval [CI], 1.12-1.29), diabetes (*P* < 0.001; OR, 18.890; 95% CI, 2.9-123.0) and chronic obstructive pulmonary disease (*P* < 0.001; OR, 7.368; 95%,CI, 1.44-37.60); among radiological features, consolidation (*P* = 0.004; OR, 5.472; 95% CI, 1.698-17.635) and peripheral GGO (*P* = 0.14; OR, 4.208; 95% CI, 1.343-13.192) were significantly correlated with fatal outcome at multivariate analysis. See Table 5 for results.

**TABLE 5:** Results of the Multivariate Analysis

	B	SE	Wald	Sign.	OR	95% CI OR	
	**1.437**	**0.583**	**6.077**	**0.014**	**4.208**	**Inferior**	**Superior**
Peripheral GGO	1.437	0.583	6.077	0.014	4.208	1.343	13.192
Consolidation	1.700	0.597	8.105	0.004	5.472	1.698	17.635
Diabetes	2.939	0.956	9.449	0.002	18.890	2.901	123.016
COPD	1.997	0.832	5.769	0.016	7.368	1.444	37.599
Age	0.187	0.034	29.713	0.000	1.206	1.128	1.290
Constant	-17.063	2.893	34.787	0.000	0.000		

Abbreviations: CI, confidence interval; COPD, chronic obstructive pulmonary disease; GGO, ground glass opacities; OR, odds ratio; SE, standard error; Sign., significance.

Lymphocytes (*P* = 0.726; OR, 0.634; 95% CI, 0.050-8.109), troponin T (*P* = 0.164; OR, 1.698; 95% CI, 0.805-3.581), C-reactive protein (*P* = 0.901; OR, 1.120; 95% CI, 0.187-6.722), D-dimer (*P* = 0.292; OR, 2.883; 95% CI, 0.403-20.632), and fibrinogen (*P* = 0.476; OR, 1.946; 95% CI, 0.312-12.118) did not show statistically significant correlation with the outcome at multivariate analysis.

Multivariate analysis using the CHAID method showed that the 2 most significant variables in predicting a fatal outcome were the age of the patient and the presence of consolidation. Mortality was 59% in patients >66 y old, and 5% in patients younger than that; in the latter group, mortality was 11% in patients presenting with consolidation, and 2% in those without it.

## DISCUSSION

In our retrospective study, we observed that the fatal outcome had a significant correlation with sex, age, various comorbidities, the assumption of ACEi, and days of hospitalization (< 0.001). The only symptom upon admission with a significant correlation was dyspnea, whereas, among blood tests, mainly troponin T, C-reactive protein, D-dimer, and fibrinogen significantly correlated with the outcome. Regarding radiological variables, consolidation, peripheral GGO, pleural effusion, and the severity score showed a significant correlation.

At the logistic regression model, only age, diabetes, chronic obstructive pulmonary disease, peripheral GGO, and consolidation showed significance as independent risk factors for fatal outcome in COVID-19 infection.

Understanding of the new coronavirus SARS-CoV-2 and COVID-19 is still evolving.

Disease severity varies broadly, with an estimated 81% of confirmed cases showing only mild disease, 14% severe pneumonia (defined as dyspnea, hypoxia, or >50% lung involvement on imaging within 24-48 h), and 5% critical disease (defined as respiratory failure, shock, or multiorgan failure).^2,15^ The case-fatality rate ranged from 0.7 to 14%, varying by location, the intensity of transmission, and the time of infection. The risk factors of mortality are still unclear: most of the fatal cases were observed in older patients or patients with underlying medical comorbidities,^15-20^ but other clinical factors still need to be identified.

In line with our results, a study on 150 patients in Wuhan, China, 31 demonstrated a significant difference in age between the dead and discharged patients (*P* < 0.001), but no difference in the sex ratio. Moreover, 63% of patients in the death group and 41% of patients in the discharge group had underlying diseases, with statistically significant differences; especially patients with cardiovascular diseases showed a significantly increased risk of death (*P* < 0.001). Significant differences between the 2 groups were also observed in white blood cell counts, absolute values of lymphocytes, platelets, albumin, total bilirubin, blood urea nitrogen, blood creatinine, myoglobin, cardiac troponin, C-reactive protein, and interleukin-6; however, the regression analysis was not performed to assess their role as independent risk factors.

Low white cells and platelet count, high D-dimer values, and high pro-thrombin time showed a correlation with mortality.^8,20^ Significantly higher levels of aspartate aminotransferase, urea, creatinine, creatinine kinase, and lactate dehydrogenase were found in dead patients than in survivors.^8,21,22^

In our study, no blood tests showed significance as an independent risk factor of mortality.

Other authors considered different variables as predictors of outcome in COVID-19 patients.

Chen et al.^4^ applied a severity score for other types of viral pneumonia, called “MuLBSTA score”^23^ to predict the risk of mortality in COVID-19 patients: this score system includes multilobar infiltration at imaging, lymphopenia, bacterial co-infection, smoking history (acute or previous), hypertension, and age (≥60 y). The authors observed that, in their population of 99 cases in Wuhan, China, the characteristics of patients who died were in line with the MuLBSTA score, but claimed the need of further studies to assess the applicability of this score in the prediction of the risk of mortality in COVID-19 patients. The evidence of age as a risk factor for unfavorable outcome is in line with our results.

In a retrospective cohort of 1590 hospitalized patients with COVID-19 throughout China,^24^ age > 65 y, and a history of coronary heart disease and cerebrovascular disease were significantly associated with nonsurvival of patients; the presence of dyspnea, high procalcitonin (> 0.5 ng/mL), and aspartate aminotransferase level (> 40 U/L) proved to be independent risk factors of fatal outcome.

From a radiological point of view, the main radiological characteristics of COVID-19 pneumonia are the alveolar disease represented by GGO (40-91%), with bilateral distribution (28-91%), and a prevalent involvement of peripheral areas (22-71%). This triad seems to be more common in the middle stages of the disease.^25,26^ These findings were confirmed to be the most common presentation in our study and a peripheral presentation was also found to be correlated to a fatal outcome at univariate analysis; this result, confirmed at multivariate analysis, suggests an underlying mechanism different from simple alveolar damage may link GGO and mortality, as shown in a work by Lang et al.^27^

The evidence at univariate analysis of a significant correlation of pleural effusion with the final outcome is in line with the results previously reported by Li et al, who suggested the role of this imaging finding as an index of severe disease.^28^

Pan et al.^25^ and Shi et al.^26^ reported that consolidation seems to be more common in the later stages; the correlation found in our work at univariate analysis between consolidation and fatal outcome can be thought to be due to patients presenting in a later stage of the disease having a worse outcome than patients presenting sooner. Nevertheless, multivariate analysis showed consolidation to be an independent predictor of mortality.

In our study, a significant correlation was found between a fatal outcome and the CXR severity score: a similar result was obtained in a retrospective study on 100 hospitalized patients with COVID-19 infection by Borghesi and Maroldi, in which the quantitative analysis of CXR, based on the type of lung abnormalities, significantly correlated with the final outcome, with severity score higher (*P* ≤ 0.002) in patients who died than those who were discharged.^29^

The value of the CXR severity score upon admission had also been proved in middle-aged patients, demonstrating that a severity score CXR score ≥ 3 was an independent predictor for intubation, and was higher in patients who died than in survivors, even if without a statistically significant difference.^30^

In our case series, most of the patients were treated with hydroxychloroquine, an analog of chloroquine with a better safety profile, and fewer drug interactions, that showed in vitro antiviral activity against SARS-CoV-2.^31^ As the epicenter of COVID-19 shifted from China to Europe, the use of hydroxychloroquine was recommended also by European authors as a possible prophylaxis and curative treatment for COVID-19^32,33^; therefore, we adopted its use in our COVID-19 treatments. The effective benefits form this treatment still remain unclear,^34^ and its use is now limited to clinical studies.^35^

This study has different limitations: first, related to the retrospective nature of this study. This is a single-center study with a relatively limited number of patients. We excluded many patients because, even if the reported symptoms were suspected for COVID-19 infection, the diagnosis was not confirmed by the RT-PCR; moreover, we excluded patients with data missing due to the retrospective nature of the study, transfer to and from other hospitals, and length of hospitalization. Even if many variables were included, according to our routine clinical practice, other variables could be assessed (ie, smoking history, other laboratory tests, and such); moreover, other possible clinical outcomes, such as the need for mechanical ventilation, can be considered.

Another limitation was that the therapy was inhomogeneous due to the absence of specific treatment guidelines at the time of the study; however, the clinical benefits of most treatments tested in COVID-19 patients remain controversial. Due to the complexity of the current clinical situation, studies including large case series and different variables are desirable and needed.

## CONCLUSIONS

Predictors of a fatal outcome in COVID-19 cases included age, the presence of underlying diseases (diabetes and chronic obstructive pulmonary disease), and the radiological evidence of peripheral GGO and consolidation.

We identified some variables associated with unfavorable outcome in patients affected by COVID-19 pneumonia. Identifying the presence of risk variables upon patient admission can improve patient management and the appropriate allocation of available health-care resources.
